# QuickCash: Secure Transfer Payment Systems

**DOI:** 10.3390/s17061376

**Published:** 2017-06-13

**Authors:** Abdulrahman Alhothaily, Arwa Alrawais, Tianyi Song, Bin Lin, Xiuzhen Cheng

**Affiliations:** 1 Department of Computer Science, The George Washington University, Washington, DC 20052, USA; hothaily@gwu.edu (A.A.); alrawais@gwu.edu (A.A.); tianyi@gwu.edu (T.S.); cheng@gwu.edu (X.C.); 2 General Department of Payment Systems, Saudi Arabian Monetary Authority, Riyadh 11169, Saudi Arabia; 3 College of Computer Engineering and Sciences, Prince Sattam bin Abdulaziz University, Al-Kharj 11942, Saudi Arabia; 4 Department of Information Science and Technology, Dalian Maritime University, Dalian 116026, China

**Keywords:** secure payment systems, QuickCash, mobile payment, P2P payment, banking, QR code

## Abstract

Payment systems play a significant role in our daily lives. They are an important driver of economic activities and a vital part of the banking infrastructure of any country. Several current payment systems focus on security and reliability but pay less attention to users’ needs and behaviors. For example, people may share their bankcards with friends or relatives to withdraw money for various reasons. This behavior can lead to a variety of privacy and security issues since the cardholder has to share a bankcard and other sensitive information such as a personal identification number (PIN). In addition, it is commonplace that cardholders may lose their cards, and may not be able to access their accounts due to various reasons. Furthermore, transferring money to an individual who has lost their bankcard and identification information is not a straightforward task. A user-friendly person-to-person payment system is urgently needed to perform secure and reliable transactions that benefit from current technological advancements. In this paper, we propose two secure fund transfer methods termed QuickCash Online and QuickCash Offline to transfer money from peer to peer using the existing banking infrastructure. Our methods provide a convenient way to transfer money quickly, and they do not require using bank cards or any identification card. Unlike other person-to-person payment systems, the proposed methods do not require the receiving entity to have a bank account, or to perform any registration procedure. We implement our QuickCash payment systems and analyze their security strengths and properties.

## 1. Introduction

The Internet of things (IoT) has been changing the fabric of banking in significant ways. The banking and credit card industries have started to respond to an increasing array of IoT opportunities and applications, especially in the payment space which plays a key part in the banking ecosystem. For a long time, payment systems have offered various methods of transferring funds between parties. Due to the increase in availability of digital technologies that supports various payment systems, societies all across the world have started using innovative micropayment products. This brings new users who have to deal with complex issues, as well as the potential to influence behaviors, privacy, and economics. However, several currently-deployed payment systems do not give enough consideration to the requirements and activities of cardholders. These systems focus mainly on providing secure payments for cardholders and banks, and ignore cardholders’ needs rooted in their behaviors and cultural practices. For example, various studies and surveys attest to the fact that many cardholders share their bank card and PIN with friends or family members. According to a recent survey conducted by Bonneaus et al. [1], 52.8% of the responders share their PINs with at least one other person, so that the person can borrow their payment cards. Sharing bankcards introduces the risk of fraud and loss of privacy. If a cardholder chooses to share her bank card and PIN with others, then the cardholder is liable for any loss incurred from misusing the card [2]. Moreover, sharing payment cards with others could increase the chance of various payment attacks such as the cloning attack. Generally speaking, it might be tempting to trust a friend or a family member, but the likelihood of fraud and payment attacks increases with untrained individuals. On the other hand, when a cardholder loses her card, there is no quick way for her to use the ATM, as replacement cards require time that could amount to several days. The proposed banking systems in this paper could help cardholders resolve these issues and allow them to access cash in just a few seconds when they lose or forget their cards.

Our suggested systems are not restricted to the aforementioned scenarios. It can also help people with limited access to banking infrastructures in some areas around the world. In many developing countries, a large percentage of individuals cannot enjoy using the ATM infrastructure because they have limited access to banking services. Recent studies show that 46% of adult men in developing countries claim that they have a bank account, while only 37% of women have a bank account. The gap is wider in Africa where 27% of men and 22% of women have banking accounts [3]. Nevertheless, according to a recent World Bank report [4], in some developing countries the number of mobile phone users exceeds the number of banking accounts. The same report indicates that about three-quarters of the world’s people now have an easier access to a mobile phone than to a bank account. Our methods would facilitate a greater access to bank ATMs for those people with mobile phones but without bank accounts. Also, the proposed systems can play a pivotal role to encourage business, donations, and fund transfers.

In this paper, we introduce two payment systems, QuickCash Online and QuickCash Offline, that allow individuals to transfer funds from their bank account to another individual. The sender can designate the amount of funds to be transferred, while the receiver does not need an account, nor must they register with a bank or any centralized payment authorizer. Nevertheless, the receiver needs to communicate directly with the sender to arrange the payment. On the other hand, nothing in the two payment systems requires the sender and the receiver to share sensitive information with each other.

QuickCash Online and QuickCash Offline are two secure ways to transfer money from person to person. QuickCash Online verifies the transaction online with the issuer using a One Time Personal Identification Number (OTP), while QuickCash Offline verifies the transaction offline at the ATM using QR code and public key cryptography. Furthermore, during the verification phase, QuickCash Offline does not require an online connection with the issuer; however, this design shifts the processing consideration from the issuer to the ATM since we need to validate the QR code. We claim that both payment systems help family members or friends send and receive money without sharing a bankcard or disclosing sensitive information such as a PIN. Furthermore, they introduce an advantage to cardholders by providing an access to ATMs during emergency cases without using a bank card. These new methods can be integrated easily with different existing banking channels such as mobile banking and online banking.

The introduction of these new payment methods would benefit individuals across a wide range of banking environments. The following are some of the advantages of the proposed payment systems:

- They enable individuals who do not have a bank account to use and benefit from ATMs without opening a bank account.
- They provide a fast and secure method to transfer money and access cash in just a few seconds.
- They help minimize the need for sharing a bankcard or sensitive information with family members or friends.
- They provide direct access to cash in cases of emergency such as losing or forgetting bankcards.
- They grant more access to banking, and makes use of limited banking infrastructures in developing countries.
- They promote the growth of transactions carried out through ATMs, thus improving their utilization.

The paper is organized as follows. Section 2 presents related work and summarizes the existing person-to-person payment systems. Section 3 and Section 4 respectively cover the designs and implementations of the QuickCash Online and QuickCash Offline payment systems. Security properties are analyzed in Section 5. We discuss the impact of IoT on payment systems in Section 6 and conclude this work in Section 7.

## 2. Current P2P Payment Systems and Related Work

**Figure 1:**
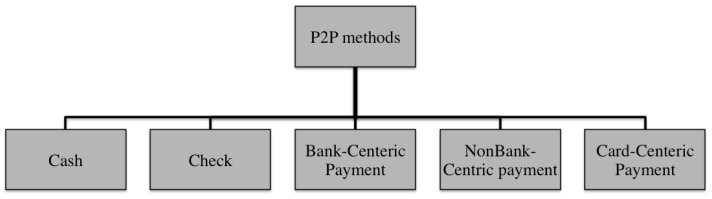
Different person-to-person payment system models.

There are several person-to-person (P2P) payment systems and this section covers the most popular ones. Note that all those mentioned in this paper are meant to be illustrative, rather than exhaustive. Figure 1 shows different payment system models to transfer fund from person to person. Most of the emerging payment systems can be categorized into three main models: nonbank-centric, bank-centric, and card-centric [5]. In the nonbank-centric model, the sender and receiver use a nonbank service such as PayPal to transfer funds to the receiver. In the bank-centric model, the individual deals with a bank directly to transfer funds from one personal account to another. The card-centric model uses a credit or debit network to transfer the payment.

### 2.1. Cash

Despite a plethora of electronic payment choices, many individuals still prefer to make P2P payments using cash. Cash payment is considered one of the most ubiquitous methods around the world. However, cash access channels are limited to face-to-face, mail, and courier services. According to the study in [6], cash is the main instrument for low-income individuals who do not have an access to alternative payment choices, or who find them expensive or hard to obtain. Cash is a convenient way to conduct small local transactions. For a large transaction, or for payment to a person in a remote location, cash is not practical.

### 2.2. Check

Check is one of the oldest forms of payments and it is widely used in the modern age. According to a recent paper [7], in 2012, about 18.3 billion checks were used for payment. The same paper shows that there was a 9.2% annual decrease in the number of written checks. Furthermore, there was 13% increase in 2012 for the checks that were converted to Automated Clearing House (ACH). Checks, however, have limitations and several factors can affect the speed of check clearing, which varies from one country to another. In general, a payer using a check has no control over when the check will be received or cleared. Moreover, check payment has many privacy concerns because the payer must reveal personal and sensitive information to the payee. Personal information such as the payer’s address and bank account number are printed on the check, and are required for check settlement.

### 2.3. PayPal

One of the most popular payment providers is PayPal [8], which is considered as an example of a nonbank-centric model. PayPal is a money transfer system used to send and receive funds online. At the end of 2014, PayPal had nearly 161.5 million active global payment accounts worldwide, up from 94.4 million in 2010 and 5 million in 2001 [9]. For a nonbank-centric model, both the payer and the payee should be enrolled in the payment service provider. Other examples of the nonbank-centric model are Google Wallet and Amazon Payments.

### 2.4. ClearXchange

ClearXchange (CXC) is a financial network in the U.S. established by Bank of America, JPM Chase, and Wells Fargo in 2011. Capital One and U.S. Bank joined ClearXchange as new owners in 2014 and 2015, respectively. The new bank members make ClearXchange one of the biggest bank-focused P2P network in the United States, reaching about 100 million online customers [10]. ClearXchange allows a member bank’s customers to send and receive P2P payments using their mobile phone numbers or email addresses. However, if a recipient is not in the consortium, the recipient is required to create a profile with clearXchange in order to complete the transaction and receive the payment [11]. Recipients from non-member banks need to provide their bank routing and account numbers during the registration, and those who do not have a bank account or do not have an address in the United States cannot use ClearXchange services. ClearXchange is a great example of a bank-centric model; other examples include Popmoney and Chase QuickPay.

### 2.5. Visa Direct

Card-centric methods play a vital role in the development of P2P payments. In a card-centric method, payment transfer is processed and cleared entirely over a debit card or credit card network. Visa Direct [12] is the most prominent example of the card-centeric payment model. It allows Visa cardholders to send payments and transfer to another eligible Visa cardholder where the transaction is processed directly over the Visa network. Using Visa Direct, a payer can perform international P2P payments to more than 170 countries, and in over 150 currencies [13]. However, Visa Direct requires both the payer and the payee to have Visa cards issued by banks belonging to the Visa network. Other card-centric P2P payment methods include MasterCard MoneySend and American Express Serve.

### 2.6. Related Work

A considerable amount of literature has been published on P2P payment and payment system verification [5,14,15]. Gao et al. proposed P2P-paid, a peer-to-peer payment method using wireless communications [16]. P2P-paid allows two users to transfer funds over Bluetooth. Monteiro et al. proposed a mobile design based on a peer-to-peer communication system for money transfer, using NFC and Bluetooth communications [17]. In [18], the authors proposed a payment design, called SmartPay,, which utilizes a privacy-preserving path discovery protocol in social networks targeting P2P mobile payments. The goal is to protect information about the relationship type, depth, and trust of the discovered paths, and to provide a bridge between social networking services and P2P payment services. Balan et al. presented the design and evaluation of mFerio, which is a near-field communication-based mobile P2P payment application [19]. The goal of designing mFerio is to replace cash-based transactions.

Another line of research is more concerned with payment system verification. Hiltgen et al. suggested two different authentication solutions for e-banking: the first one is based on short-time passwords and the second on certificates [20]. The authors in [21] (extended version appears in [22]) proposed a new cardholder verification method for card payments using a multi possession-factor authentication with a distance bounding technique. In [23], the authors proposed using smartphones and location verification for payments, and the authors in [24] suggested leveraging cellular infrastructures to mitigate frauds.

All the aforementioned payment systems require both the sender and the receiver to have an account or to register with a third party service. The two proposed payment systems in this paper do not require a recipient to have an account, or to register with a bank or non-bank service. Nevertheless, they provide quick and convenient access to cash.

## 3. QuickCash Online

**Figure 2:**
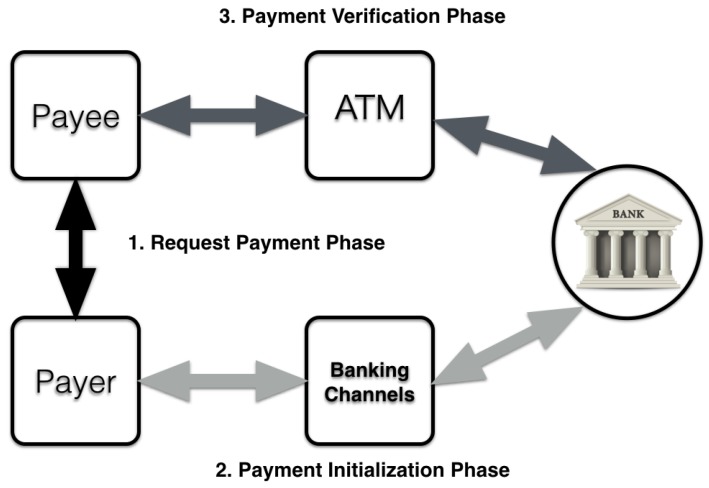
The transaction flow.

In this section, we describe the proposed QuickCash Online payment system, which consists of three major phases depicted in Figure 2 and involves four main entities: the payer, the payee, the ATM, and the bank host system. The ATM is a payment terminal and is connected to the host bank. The host bank issues virtual accounts and maintains the associated information used in the payment verification phase. For convenience, this paper refers to the payer in the proposed payment system as “she” and to the payee as “he”. Table 1 lists the abbreviations that are used in describing the proposed payment system.

### 3.1. QuickCash Online Description

The first phase is the payment request, which involves the payer and the payee. In this phase, the payee should contact the payer and request the payment. The payee should designate the amount of fund and provide three important pieces of information: the location of the ATM (ATML) to withdraw the fund, the specific time for cashing the payment (Transaction Validity Period TVP), and a mobile phone number *m*. The location can be the physical address of an automatic teller machine ATM, and the time can be a specific range of hours and a start time such as two hours after 6:00 PM. A mobile phone number should be associated with the virtual account VA created for the transaction by the bank. It is used to receive a One-time Personal Identification Number OTP during the verification process in the last phase.

The second phase is for transaction creation, and it involves the payer and the bank. The payer uses all the provided information from the payee to create a transfer transaction. She should login to her account using online banking or any mobile banking application. Once the transaction is created, a 16-digit random virtual account number is generated to identify the transaction. The payer then provides the payee with this number via a secure channel.

The last phase is cashing the payment, and it includes the payee, the ATM, and the bank host system. The payee should use the 16-digit virtual account number that was generated by the issuing bank. To cash the transaction, he should go to the designated automatic teller machine during the agreed time period, enter the 16-digit virtual account number at the ATM, and then request that the specified amount of money be reserved against the payer’s fund. The ATM forwards the request through the banking network to the bank that issued the virtual account number (called the issuing bank).

The ATM along with the bank host system verify the identity of the payee using the associated mobile phone number and the virtual account number. If the virtual account number is valid, the issuing bank sends an OTP to the payee, sending the eligibility response back through the network to the ATM. If not, the issuing bank denies the transaction and sends a negative response to the ATM. For transaction verification, the bank host system compares the OTP entered by the payee with the one sent to the mobile phone that was bound to the virtual account number during the transaction creation phase.

It is important to note that the transaction is valid for a designated location and a designated period of time, and it expires at the end of that time. Transaction validity period is a mechanism that limits the lifespan or lifetime of a transaction in the proposed payment systems. It is used to provide an extra level of protection, and gives the payer an additional level of control. The implementation of a transaction validity period can be handled in several ways such as a counter in hours and minutes, or a timestamp attached to the transaction. If the prescribed event count or timespan elapses and the transaction is not cashed, then the transaction is discarded, and the reserved fund returns to the payer’s account. In the following we detail the three phases.

#### 3.1.1. First Phase of QuickCash Online: Request Payment

For transaction security and payer control, the proposed system depends on three essential factors: a transaction location control factor, a transaction validity period control factor, and a possession factor. The payee should provide the following information to the payer:

- Automatic Teller Machine’s Location (ATML) as a location factor.
- Transaction Validity Period as a time factor (TVP).
- Mobile Number *m* as a possession factor.

These three factors are essential to verify the transaction during the withdrawal phase.

#### 3.1.2. Second Phase of QuickCash Online: Payment Initialization

After completing the first phase and having the required information from the payee, the payer is ready to create the transaction using various banking channels such as mobile banking or online banking. The payer should login to her bank and select QuickCash transfer option. Then, the recipient information such as the mobile number and the name should be entered in the specified fields. The payer should designate the fund, the ATM location, and the lifespan of the transaction based on the information received from the payee.

**Figure 3:**
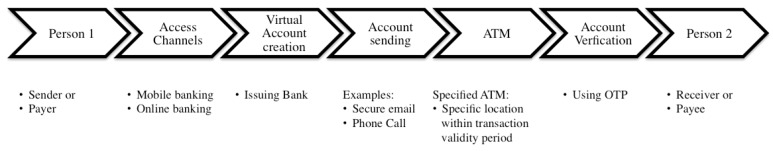
QuickCash Online transaction life cycle.

After completing the payment creation step, the payer should provide the payee with a virtual account number, which consists of a16-digit number. The transfer of the virtual account could be done in several ways, such as a phone call, a secure Short Message Service or a secure email. The transaction life cycle, from the payer to the payee, is presented in Figure 3.

It is worth mentioning that we choose the16-digit account number because it is commonly used and widely accepted.

#### 3.1.3. Third Phase of QuickCash Online: Withdrawal Process

**Figure 4:**
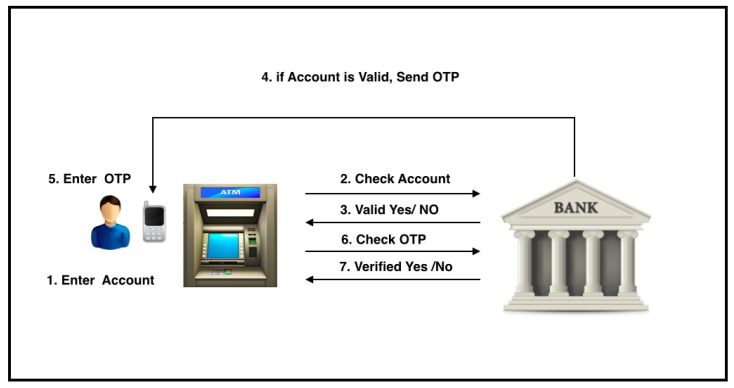
Payment verification phase in QuickCash Online.

Transaction verification is a crucial part of the withdrawal process in QuickCash Online, which is detailed as follows and illustrated in Figure 4.

1. The payee should enter the received virtual account number VA at the specified ATM within the transaction validity period.
  R→ATM:{VA}
2. After entering the virtual account number, the ATM sends an identification request containing the VA and its location to the bank host through the banking network.
  ATM→BANK:{VA,ATML}
3. The bank host identifies the request and checks the validity of the transaction, verifies the transaction location, and checks whether the number of verification attempts has reached MaxAttempts. In QuickCash Online, we allow MaxAttempts to prevent brute force attacks before the transaction is revoked.
4. The bank host sends the result of the identification to the ATM. If identified; the bank host sends OTP to the user for virtual account holder verification; otherwise, the bank host instructs the ATM to show an error message.
  BANK→ATM:{Identification_Result}
  BANK→R:{OTP}<Ifidentified>
5. The ATM instructs the payee to input the OTP.
  R→ATM:{OTP}
6. The ATM sends the entered OTP to the bank host for PIN verification
  ATM→BANK:{Entered_OTP}
  If verified (Entered_OTP = Sent_OTP), then the bank host sends a Verified message to the ATM. Otherwise, the bank host sends a Not Verified message. Algorithm 1 shows a high-level description of QuickCash Online verification using the three verification factors ATML, TVP, and OTP.
  BANK→ATM:{Verification_Result}
7. If a Verified message is sent, the bank posts debit to the payer’s account.
8. In addition, the bank sends an email or text message to the payer *S* informing her that the payee has cashed the payment.
  BANK→S:{Payment_Confirmation}

**Algorithm 1:** QuickCash Online Verification.
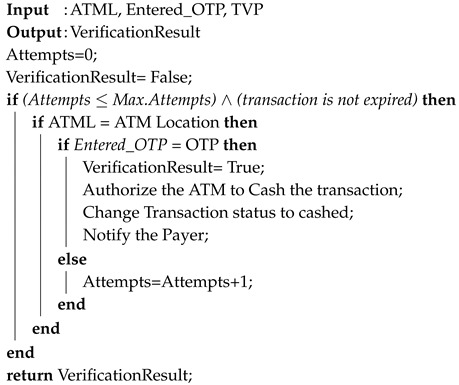


QuickCash Online does not require any hardware modification at the ATMs; it only requires some software modifications in the banking side. The first modification should be at the ATM level. ATM interface (ATM menu) should include an option to support QuickCash payment. The second change requires the banking system to implement the main requirements of the QuickCash system such as virtual account issuance and management. Additionally, the banking system should have virtual account verification capabilities using OTP. Finally, the changes should be made in the online and mobile banking channels by adding the QuickCash Online option.

### 3.2. Experiment Details and Results

To show the practicality and measure the authentication performance of QuickCash Online, we built an authentication server and an ATM. The authentication server maintains virtual accounts along with the verification information such as the ATM location, transaction validity period, and the bound mobile phone number. In addition, the authentication server is connected to a messaging service provider named Twilio [25], which provides text message services. The authentication server utilizes the Twilio Application Programming Interface to send randomly generated OTPs to the registered user during the verification process. The authentication server is implemented in JAVA version 1.8.025-b17 and runs over Macbook Pro with OS X Yosemite version 10.10.2. The hosting machine runs a processor 1.8 GHz Intel core i5 with a memory of size 4 GB 1600 MHz DDR3. The ATM simulator is implemented in JAVA version 1.8.0-25 and run over a Mac air with the OS X Yosemite version 10.10.2 as well. The hosting machine for the ATM simulator runs a processor 1.8 GHz Intel core i5 with a memory of size 8 GB 1600 MHz DDR3.

**Figure 5:**
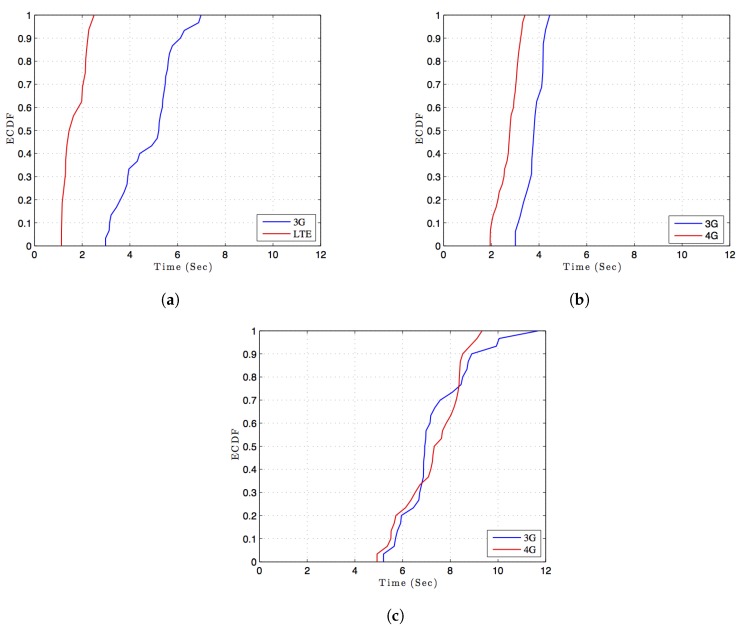
The empirical CDFs of the time it takes to receive an OTP: (**a**) Local operator A; (**b**) Local operator B; (**c**) International.

We performed a test for 3G, 4G, and LTE using two local cellular operators and one international cellular operator. For each connection setting, we measured the OTP time, i.e., how long it takes from the moment the ATM issues a request until the moment the payee receives the OTP. For more accurate results, the experiment was repeated 30 times for each connection setting. The empirical CDFs of the time measurements were plotted in Figure 5. The results show longer OTP times when using international cellular operators. This behavior is presumably caused by the time it takes to send the OTP message from the international cellular network to the local roaming network. On the other hand, the LTE technology provides the best verification results with an average of 1.57 s. All results are summarized in Table 2.

## 4. QuickCash Offline

**Figure 6:**
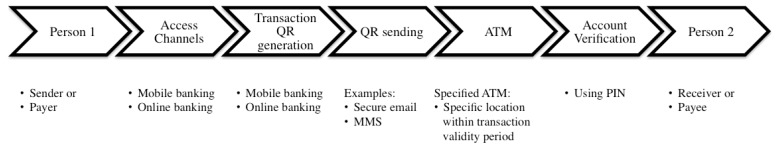
QuickCash Offline transaction life cycle.

In this section, we explain cryptographic primitives and QR codes which play a crucial role in QuickCash Offline. We then describe the technical details of QuickCash Offline and go through the transaction life cycle that is presented in Figure 6. Finally, we introduce our experimental results to show the efficiency of our method.

### 4.1. Cryptographic Primitives

Cryptography is paramount in the design of QuickCash Offline, we rely on many cryptographic mechanisms such as public key encryption, cryptographic hash function, and digital signature. All these mechanisms are used to provide essential security services which include integrity, authentication, and non-repudiation.

Public key cryptography or asymmetric cryptography provides confidentiality and message authentication, which are two essential security services in our design. In a public key cryptography system, there are two related keys: a private key, and a public key. The former should be stored securely and the latter is published to the world [26]. In our design, we use RSA cryptosystem since it is widely used for secure data transmission in the banking sector.

**Figure 7:**
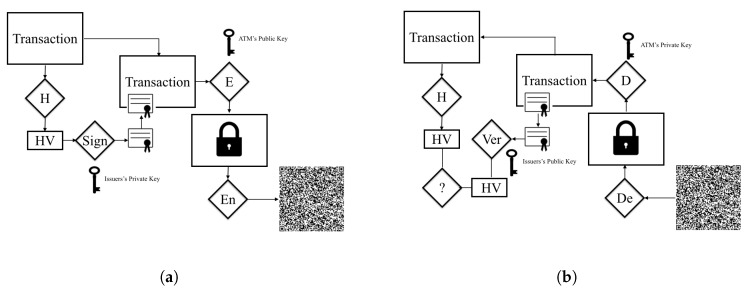
Simplified examples of generating and verifying QuickCash Offline transactions. The transaction information and the corresponding PIN in a form of QR code are illustrated in (**a**). After the transaction is composed, the hash value HV is generated. Then, the hash value HV is encrypted with the private key of the issuing bank to create the digital signature on the transaction T. The whole transaction along with the digital signature is encrypted using the ATM’s public key. After that, the encrypted result is fed to the QR encoder algorithm (EN) to generate the QR code. (**b**) shows the verification procedures. The ATM scans the QR code and decodes it using the decoder algorithm (De). The result of the decoder is fed to the decryption algorithm to be decrypted using the ATM’s private key. Then, the ATM verifies the signature by comparing the hash value HV using the issuing bank’s public key. The transaction is valid when the computed signature matches the received one.

Hashing is a technique to produce fixed data size, called digest from a variable-length message M by using a hashing algorithm. The goal of using hashing is to ensure integrity. Any change to any bit in M should result a change to the hashing algorithm output [27]. Hashing and digital signatures are used in a broad range of banking applications and payment protocols. In our design, the hashing algorithm output is encrypted with an issuer’s private key. The ATM can verify the integrity of the transaction that is associated with the digital signature using the issuer’s public key. Figure 7 shows, in a simplified fashion, how a hash code and public key cryptography are used to provide a digital signature in QuickCash Offline.

### 4.2. QR Codes

A QR code stands for a Quick Response code, which is simply a two-dimensional barcode. However, a QR code can hold more information than a standard one-dimensional barcode. It was invented in 1994 by Denso Wave for the automotive industry in Japan. Recently, QR codes became widely used in various disciplines such as marketing, item tracking, document verification, and social media. The amount of information that can be encoded into the QR code depends on the size of the matrix, the data type, and the error correction level. The matrix can handle up to 7089 characters if the data type is numeric only, and up to 4296 characters for both numeric and letters [28]. QR codes provide a convenient and fast way to store and send data.

Currently, QR Codes have 40 versions ranging from 1 to 40, and the size of each version is different. The size of QR code depends on the horizontal and vertical dimensions of the QR version used. For example, version 1 consists of 21 modules for each side, where the module can be black or white square. For each increase in version number, there is an increase in the size of the two physical dimensions by 4 modules. This means that version 2, version 3 and version 4 have 25×25, 29×29, and 33×33 modules, respectively. The most capacious QR code version is version 40, which has 177 columns and 177 rows. This large number of columns and rows allow one to have 31,329 modules in total.

In a typical QR system, there are two computing ends: the first end produces the QR codes and the second one consumes them. The producing end should have an encoding software to convert the data to a QR code. On the other hand, to decode a QR code, the decoding device should have a reader software and a camera. The user of QR codes should place the QR code in front of the camera. The reader software can automatically take and decode the picture. QR codes have been well defined and published as an ISO standard with number ISO/IEC 18004:2015 [29].

### 4.3. QuickCash Offline Description

In this subsection, we detail the QuickCash Offline, which also contains 3 phases. The first phase is similar to that of the QuickCash Online. The payee should contact the payer and request the payment. Moreover, The payee should designate the amount of the fund and provide the location of the ATM and a specific time for cashing the payment. In the following we describe the second and third phases.

#### 4.3.1. Second Phase of QuickCash Offline: Payment Initialization

The second phase is for transaction creation, which involves the payer and the bank. The payer uses all the provided information from the payee to create a transfer transaction. In this phase the payer should login to her account using online banking or mobile banking applications. Once the transaction is created, the bank issuer should push the transaction information directly to the specified ATM via the secure banking network. In addition, the payer should provide the payee with the generated QR code and the PIN. The following steps summarize the payment initialization procedure:

1. The payer creates a transaction that contains the following information:
  - Virtual account number VA.
  - Amount of the transaction.
  - ATM Location ATML.
  - Transaction Validity Period TVP.
  - PIN.
2. The whole transaction is hashed using one way hashing function h(.), and the hash digest is signed by the bank using the bank’s private key.
3. The transaction along with the digital signature is encrypted by the bank using the ATM’s public key.
4. The QR code of the whole encrypted transaction is generated by the bank using a QR encoder algorithm.
5. The payer securely sends the QR code along with the PIN to the payee.

Note that the above procedure assumes that the bank can sign and encrypt the transaction and compute the QR code on behalf of the payer, which is reasonable as the payer registers her account with the bank. By this way we do not require a payer to have the computational power suitable for public key cryptography, which makes QuickCash Offline a viable approach for the general public.

In addition, note that after completing the payment creation step, the payee should receive from the payer the QR code and the PIN. The transfer of the QR code could be done in several ways such as a secure Multimedia Messaging Service or a secure email.

#### 4.3.2. Third Phase of QuickCash Offline: Withdrawal Process

**Figure 8:**
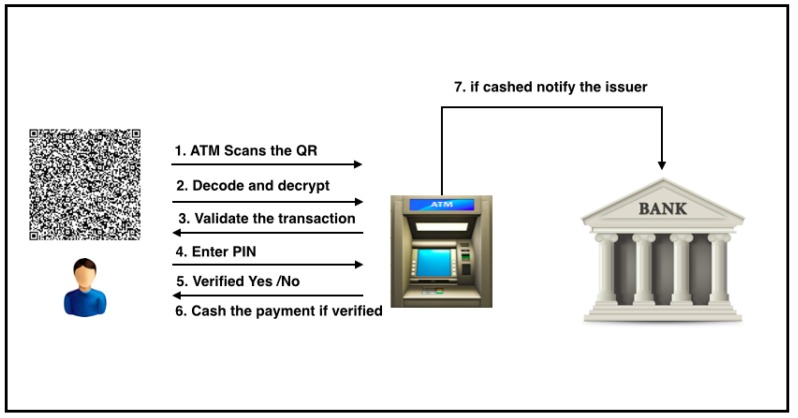
The payment verification phase in QuickCash Offline.

The following provides a detailed, step-by-step explanation of the payment withdrawal process using QuickCash Offline. These steps are also illustrated in Figure 8.

1. The payee should have the QR ready to be scanned at the designated ATM within the transaction validity period.
2. The scanned QR code is decoded and converted to the message E.
3. The ATM decrypts the encrypted message E using the ATM’s private key. The message contains two parts: the transaction details, and the digital signature.
4. The ATM identifies the transaction and checks the transaction validity via signature verification.
5. If the transaction is identified and valid, and number of verification attempts is less than or equal to the maximum allowable attempts, then the ATM prompts the user to enter the PIN.
6. If the user is verified (entered PIN = PIN), then the ATM allows the payee to access the cash, and then sends a cashed transaction message to the bank. Algorithm 2 illustrates a high-level description of QuickCash Offline verification.
7. The bank posts debit to the payer account and sends an email or text message to the payer informing her that the payee has cashed the payment.

**Algorithm 2:** Offline QuickCash Verification.
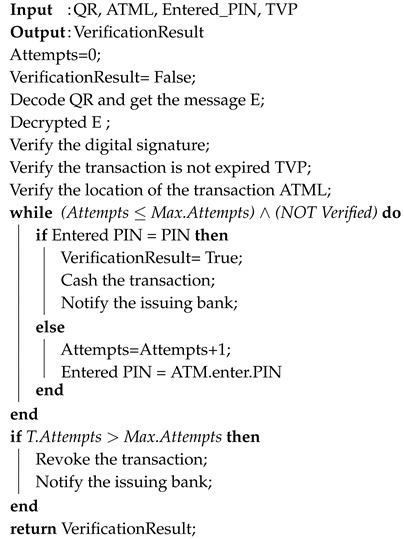


### 4.4. Experiment Details and Results

We built a prototype to generate and verify QuickCash Offline transactions. QR codes are generated using a low-level correction code, and encrypted using an RSA algorithm with a 2048-bit key. The encrypted QR codes consist of the virtual account number, the ATM location, the transaction validity information, the PIN, and the digital signature.

**Figure 9:**
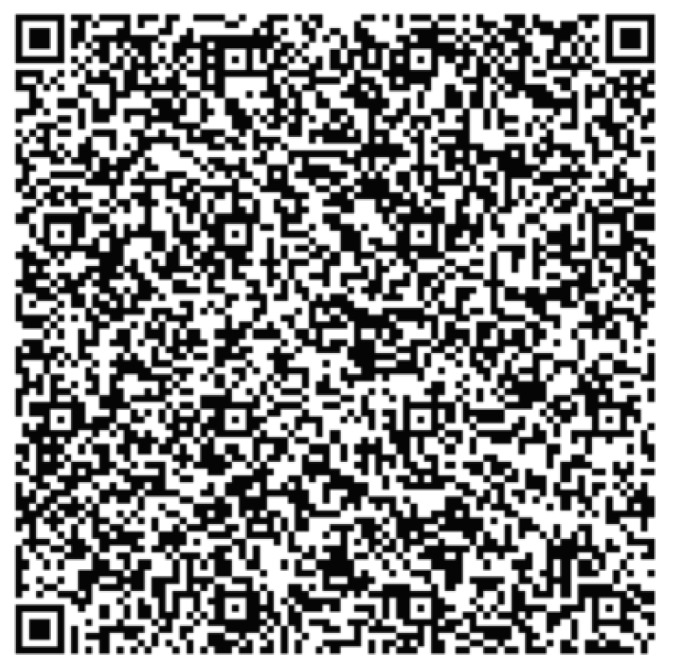
An example of a QR code that is generated by QuickCash Offline.

QuickCash Offline requires the ATM to be equipped with a camera similar to the mobile phone camera. The goal of using camera technology is to capture the QR codes that can be processed by the ATM. Figure 9 illustrates a QR Code that represents a P2P transaction using QuickCash Offline.

To understand how quickly an ATM can read and decode a QR code in QuickCash Offline, we quantified the time to read a QR code using different mobile phone cameras. In our experiment, we used Apple iPhone 4, iPhone 5, and iPhone 6, which have different screen sizes, and repeated each test 30 times to get the averaged result. From the experiments, it can be clearly seen that the distance between the QR code and the camera can affect the size of the QR code on smartphones. In addition, the precision of the camera and the processing power in the smartphones affect the reading time.

**Figure 10:**
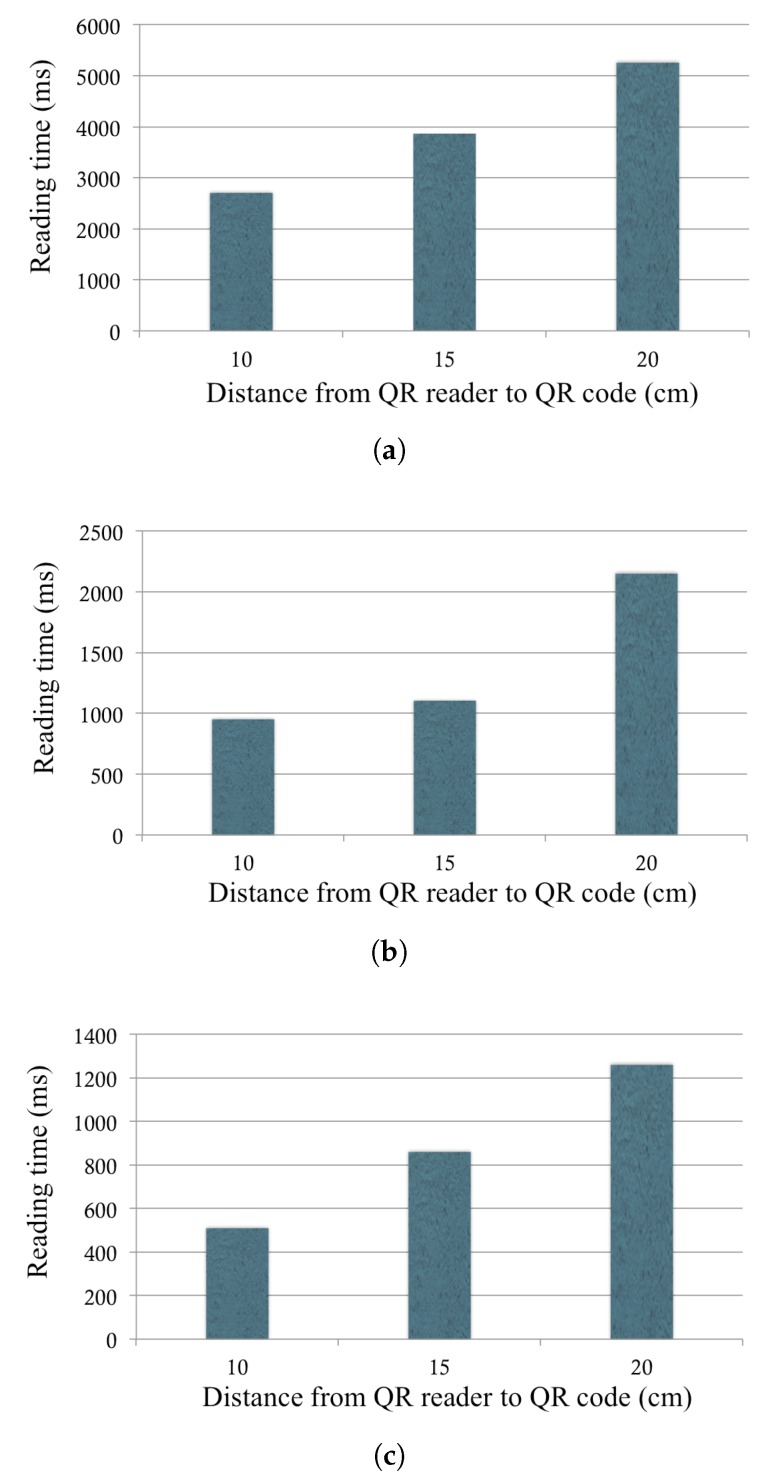
The time to read a QuickCash Offline transaction: (**a**) The reading time for iPhone 4; (**b**) The reading time for iPhone 5; (**c**) The reading time for iPhone 6.

From the results shown in Figure 10, we observe that all smartphones can read QR codes very quickly. The average time for QR reading is 3950, 1360, and 870 ms for iPhone 4, iPhone 5, and iPhone 6, respectively. The best performance occurs when the distance is 10 cm between the QR code and the rare camera. These results support the practicality of integrating QuickCash Offline into ATMs.

## 5. Security Analysis

In this section, we analyze the security strengths of our proposed person-to-person payment systems and explain how they can counter various attacks. Assume that the link between the ATM and the bank is encrypted using a standard encryption, and that the authentication server at the bank is secured and fully compliant with the Payment Card Industry Data Security Standard (PCI DSS) requirements [30].

### 5.1. Privacy

QuickCash Online and QuickCash Offline can tackle many privacy issues for both the payee and the payer. The payer does not have to share any sensitive information such as a bank account (as the case in check or bank cards), or a PIN (as the case in sharing behavior reported in [1,31,32]). Furthermore, the payee is not required to disclose any sensitive information such as a bank account or perform any registration to receive a payment. The proposed design is privacy-friendly, and it can help mitigate sharing behaviors and protect users from further bad consequences such as losses and frauds.

### 5.2. Brute-Force Attacks

A brute-force attack attempts every possible combination for a password in order to gain an access to a system. A brute-force attack is not an effective attack against QuickCash Online OTP for various reasons. In order to execute the protocol correctly, the recipient has to know three different pieces of information: the virtual account, the location of the ATM, and the validation period of the transaction. This information should be known only by the payer, the payee, and the issuing bank. However, in case when an attacker is able to know all the detailed information of a specific transaction, there is still another line of defense in limiting the number of attempts: the total number of attempts is restricted to a maximum allowable number of attempts such as 7, which means that when the number of attempts exceeds 7, the transaction is revoked, and the fund is returned to the payer’s account.

QuickCash Offline relies on standard public key cryptography, RSA with 2048-bit key, and password hashing that provides adequate security. Without a doubt, the use of a strong password can improve security, but there should be a balance between security and usability.

### 5.3. Verification

Verification is used to check whether the recipient holding the payment instrument at the ATM is the legitimate owner of the instrument. For authentication in QuickCash Online, we utilize the ATM location, the transaction validity period, and the virtual account number in addition to the OTP which is used in many authentication schemes [33,34,35,36]. The recipient’s mobile phone is bound to the transaction, and it plays a crucial role in the verification process.These factors provide an adequate authentication level for QuickCash Online. For QuickCash Offline, the user should know not only the knowledge factors of the ATM location, the transaction validity period, and the PIN, but also the QR code known only by the user.

### 5.4. Integrity

Transaction integrity ensures the accuracy and completeness of a transaction over its entire transaction life cycle. This means that the transaction cannot be altered in an unauthorized way or undetected manner. QuickCash Online integrity is maintained by the issuing bank, and once the transaction is issued, there is no way to modify the transactions without the payer’s approval. The integrity of the transactions in QucikCash offline is maintained by the use of the digital signature. The designated ATM can verify the signature and detect any modification.

### 5.5. Theft of the Virtual Account or QR Code

Losing the registered phone does not pose a huge risk, since the thief requires additional verification information such as the virtual account, the location of the ATM, and the transaction validity period, to cash the transaction. Video surveillance and security cameras at ATMs can help detect thieves and prevent fraudulent use of the stolen phones. Furthermore, the theft of a virtual account in QuickCash Online does not provide an advantage for an attacker because the mobile phone and OTP are still required. Also, it requires an advanced knowledge about other verification information such as the ATM location and the valid period of the transaction. On the other hand, QuickCash Offline is basically a two-factor authentication payment system that requires both an encrypted QR Code and a strong password; thus it is not vulnerable to theft of a QR Code.

### 5.6. Double Spending

Double spending is the result of cashing a transaction more than once. It can be prevented using different mechanisms, and is relatively easy to prevent in online environments. QuickCash Online protects against double spending by verifying each transaction at the issuing bank; after the transaction is cashed, there is no way to spend the transaction again. In QuickCash Offline, double spending can be prevented by centrally handling a transaction book that tracks the status of the transactions.

## 6. QuickCash and IoT

The Internet of Things provides tremendous opportunities for various P2P applications, including our proposed payment systems. As we have discussed earlier, verification plays a crucial role in the design; it is used to check whether the recipient holding the payment instrument at the ATM is its legitimate owner. Other than the recipient’s mobile phone, any personal IoT device can be bound to the transaction and play a key part in the verification process. The idea of using things for verification process is not new; it has actually been widely adopted for its convenience and effectiveness [23,37].

We believe it will be common for users to own multiple Internet of Things devices and access to multiple cloud computing services and platforms in the near future. Our proposed P2P payment systems can be implemented using IoT devices such as a smartwatch. For example, with a connected personal IoT device that uses a shared IoT platform with the issuing bank, a payee would be alerted when the ATM’s location is near, or when the transaction is about to expire, or if the designated ATM is out of cash. With this information in hand, the payee could order a new transaction from the payer or request an increase in the transaction validity period, “with the push of a button”.

Furthermore, the capability for sensing could create a fertile ground for the development of QuickCash. In our design, a user enters a virtual account or scans a QR code using a scanner attached to the ATM. However, the virtual account or the QR code can be bound to a personal IoT device, and the ATM could automatically recognize the IoT device that is touching, by taking advantage of distinctive electromagnetic noise emitted by such devices [37] or acoustics-based technology [38] to identify the transaction. In fact, using a series of sensors, an ATM can gain access to the transaction information upon a payee’s arrival at the cash machine. The IoT obviously can make it easy for users to simply walk in, touch the ATM, and walk out with cash.

## 7. Discussion and Conclusions

Existing payment systems such as [8,12,16] require both the sender and the receiver to have an account or to register with a third party service. In this paper, we introduced and analyzed two P2P payment systems: QuickCash Online and QuickCash Offline, which not only facilitate fund transfer, but also act as a second channel for cardholders to withdraw money from ATMs. The use of this second channel could help provide a solution for family members and friends to withdraw funds instead of sharing the actual card. It can also be used in emergency situations such as the case when cardholders lose their bank cards. Furthermore, both payment systems can help increase unbanked users’ access to the banking infrastructure (such as ATMs) in developing countries.

Our proposed payment systems utilize the existing banking infrastructure, the ATM, and make P2P payment accessible by the widest possible community of users. In addition, this new payment model could support economic growth in developing countries and create new opportunities. We implemented both payment systems to demonstrate their feasibility and potential in real-world banking for the transfer of micro funds. Both payment systems provide payers with easy ways to transfer funds, payees with quick access to cash, and both with stronger security and improved privacy. Apparently, our proposed payment systems are very practical and can solve many privacy issues with sharing behaviors. They also provide a desirable option for micro-merchants such as babysitters, tutors, and gardeners, or any service providers who prefer quick access to cash.

There exist several directions that can be further explored in our future research. First of all, our plan is to test QuickCash Offline on smart watches such as the Apple Watch and the Samsung Galaxy Gear. Second, we plan to investigate the design of international P2P payment systems that provide quick access to cash utilizing the world’s banking infrastructure. In addition, we will study techniques to improve authentication such as leveraging IoT devices and utilizing visual decryption and visual signature verification. Finally, reporting on usability and user studies of the proposed P2P payment systems should be further investigated in our future research.

## Figures and Tables

**Table 1:** Abbreviation and description.

Abbreviation	Description
*S*	The individual who sends the payment
*R*	The individual who receives the payment
ATM	Automatic Teller Machine
ATML	Automatic Teller Machine Location
TVP	Transaction Validity Period
VA	Virtual Account
PIN	Personal Identification Number
OTP	One Time PIN
*m*	The mobile phone number of the payee

**Table 2:** Time for OTP verification during payment transactions.

	Local Operator A		Local Operator B		International Operator	
	3G	LTE	3G	4G	3G	4G
Average	4.85	1.57	3.79	2.75	7.39	7.33
Min	2.98	1.03	3.01	1.95	5.20	4.93
Max	6.98	2.50	5.01	3.41	11.69	9.33
Std dev	1.13	0.45	0.48	0.43	1.46	1.23
